# Advances in Treatment of Refractory Advanced and Recurrent Cervical Adenocarcinoma: Molecular Heterogeneity and Differences from Squamous Cell Carcinoma

**DOI:** 10.3390/ijms27198645

**Published:** 2026-09-27

**Authors:** Yusuke Taira, Wataru Kudaka, Yuko Shimoji, Tomoko Nakamoto, Yoshihisa Arakaki, Takuma Oyama, Akihiro Yoshida, Toshiaki Watanabe, Hina Akamine, Ikuko Komesu, Masayuki Sekine

**Affiliations:** Department of Obstetrics and Gynecology, Graduate School of Medical Science, University of the Ryukyus, 1076 Kiyuna, Ginowan, Okinawa 901-2720, Japan; h115474@cs.u-ryukyu.ac.jp (Y.T.);

**Keywords:** cervical cancer, cervical adenocarcinoma, clinical trials, molecular-targeted agents, immune checkpoint inhibitor, antibody–drug conjugate

## Abstract

Treatment for locally advanced and recurrent cervical cancer has advanced substantially with immune checkpoint inhibitors, induction chemotherapy, bevacizumab, and antibody-drug conjugates. However, subgroup analyses of major phase III trials suggest that cervical adenocarcinoma (AC) may respond differently from squamous cell carcinoma (SCC). Therefore, novel treatment strategies based on the distinct biological characteristics of AC are needed. This review aimed to evaluate the efficacy of current treatments for AC by examining histology-specific subgroup analyses from major clinical trials and to identify potential therapeutic strategies for AC. We also reviewed ongoing AC-focused clinical trials of novel molecularly targeted agents and combination therapies for advanced or recurrent disease. A structured search of PubMed and ClinicalTrials.gov through 1 June 2026, identified studies of advanced or recurrent cervical adenocarcinoma. Randomized trials, prospective studies, and histology-specific subgroup analyses were included, while case reports, preclinical studies, and SCC-only studies were excluded. Survival and response outcomes were extracted, with histology-specific findings considered exploratory. AC exhibits distinct molecular and immunological features compared with SCC, including frequent alterations in *ERBB2/3*, *KRAS*, *PIK3CA*, *PTEN*, *ARID1A*, and *STK11* and a relatively immune-cold tumor microenvironment. HPV-independent AC, particularly gastric-type adenocarcinoma, shows aggressive clinical behavior and resistance to conventional therapies. Although bevacizumab, immune checkpoint inhibitors, and tisotumab vedotin have improved outcomes in cervical cancer, evidence specific to AC remains limited because most pivotal trials predominantly enrolled SCC or reported combined AC/adenosquamous subgroups. Emerging strategies targeting HER2, CLDN18.2, TROP2, Nectin-4, FAK, PI3K/AKT/mTOR, and ROS1 may provide new therapeutic opportunities for AC. AC represents a biologically heterogeneous disease distinct from SCC, supporting treatment strategies based on histology and molecular characteristics. Future AC-specific clinical trials and molecular stratification according to HPV association, histological subtype, and actionable alterations are needed to establish biomarker-driven personalized therapy, particularly for treatment-resistant HPV-independent AC.

## 1. Introduction

While cervical squamous cell carcinoma (SCC) has been declining due to the widespread use of HPV vaccines, cervical adenocarcinoma (AC), which is associated with HPV to a relatively lesser extent, is on the rise [1]. Currently, AC accounts for approximately 20–30% of cervical cancers, and the increase is particularly pronounced among younger women. Whereas SCC is primarily associated with HPV type 16, AC is strongly associated with HPV type 18 [2], and because it originates within the cervical canal, early detection via cytology is difficult [3]. Furthermore, AC has a high capacity for invasion and metastasis, and is particularly prone to lymph node and hematogenous metastasis. Additionally, its low radiosensitivity contributes to a poor prognosis [4]. Since the majority of cervical cancers are SCC, many clinical trials analyze SCC and AC together as a single group. However, as mentioned earlier, because SCC and AC differ in their biological and clinical characteristics, analyzing them together carries the risk of overlooking important findings. While some clinical trials have conducted analyses stratified by histological type—using SCC and AC as stratification factors—such trials are few in number. Even when examining the standard treatment guidelines of various countries, the current National Comprehensive Cancer Network (NCCN) and the European Society for Medical Oncology (ESMO) guidelines do not clearly specify treatment strategies for each histological type by classifying SCC and AC separately [5,6].

In recent years, it has been reported that genetic and epigenetic differences exist for each histological type of cervical cancer, relating to mechanisms of development, patterns of proliferation and progression, biomarkers of drug sensitivity, and mechanisms of drug resistance; these differences are thought to contribute to variations in treatment response and prognosis. This review article focuses on the molecular findings and results of clinical trials to date regarding AC, with the aim of proposing optimal treatment strategies for this disease. Specifically, we will extract the results of subgroup analyses from the clinical trials reported to date and compare the results to propose better treatment options for patients with AC. In addition, we would like to review the details of new ongoing clinical trials specifically for advanced and recurrent patients with AC, including new molecular-targeted agents and, in particular, combinations of molecular-targeted agents.

This narrative review used the following literature search protocol. A structured literature search was conducted using PubMed and ClinicalTrials.gov from database inception through 1 June 2026, to identify clinical studies evaluating treatments for locally advanced, persistent, recurrent, or metastatic cervical adenocarcinoma (AC). PubMed was searched using combinations of the terms “cervical cancer,” “cervical adenocarcinoma,” “advanced,” “recurrent,” “metastatic,” “persistent,” “chemotherapy,” “immunotherapy,” “immune checkpoint inhibitor,” “targeted therapy,” and “antibody-drug conjugate.” Supplementary searches were performed using pivotal trial and drug names, including GOG-240, KEYNOTE-826, KEYNOTE-A18, EMPOWER-Cervical 1, BEATcc, innovaTV 301, INTERLACE, pembrolizumab, cemiplimab, bevacizumab, tisotumab vedotin, and trastuzumab deruxtecan. Reference lists and supplementary appendices of relevant articles were also reviewed. ClinicalTrials.gov was searched to identify ongoing trials of novel targeted therapies, immunotherapies, and antibody-drug conjugates. Randomized trials, prospective phase I/II studies, and histology-specific subgroup analyses reporting outcomes for AC or adenocarcinoma/adenosquamous carcinoma were included. Case reports, preclinical studies, and studies exclusively involving squamous cell carcinoma were excluded. Overall survival, progression-free survival, objective response rate, and hazard ratios were extracted when available. Because most trials were not powered to detect histology-specific treatment effects, subgroup findings were interpreted as exploratory. OpenAI’s ChatGPT 5.5 was used to create the figure. The authors provided instructions regarding the figure’s composition, the molecular biological pathways to be included, therapeutic targets, and drug mechanisms of action, using ChatGPT to generate the schematic diagram. The authors then cross-checked the generated figure against relevant literature, verified the scientific content, and made necessary revisions.

## 2. Etiology and Epidemiology

### 2.1. Differences in Pathogenesis from Molecular Perspective Between SCC and AC

The two major histological subtypes of cervical cancer, SCC and AC, exhibit distinct molecular and immunological profiles. Multiple studies have identified significant differences in gene expression patterns between these subtypes. However, the risk factors for both SCC and AC are largely the same. Specifically, these include: (1) having multiple sexual partners; (2) early age at first sexual intercourse; (3) a high number of pregnancies and deliveries; and (4) use of oral contraceptives. Epidemiologically, the risk of developing AC varies by region and ethnicity, with higher incidence rates observed in Asia. AC tends to develop at a younger age than SCC, and high serum progesterone levels are considered a risk factor [7,8].

HPV types 16 or 18 are detected in 74% of SCC cases and 78% of AC cases [9], however, the positivity rate for HPV type 18 is higher in AC than in SCC [10]. According to a report by Pirog et al., when HPV types 18, 16, and 45 are considered together, infection with these three subtypes accounts for 94.1% of AC patients [2]. Even within AC, the HPV infection rate varies by pathological subtype; the infection rate for endometrioid carcinoma, serous carcinoma, clear cell carcinoma, and gastric-type adenocarcinoma (GAS) ranges from 8.3% to 27.3%, and it is well known that the infection rate is particularly low in GAS. Taguchi et al. reported that HPV type 18-positive AC patients are characterized by a high detection rate in high-grade adenocarcinomas and small cell carcinomas, whereas the detection rate is low in patients with precancerous lesions; in this respect, it differs from other high-risk HPV types [11]. Furthermore, HPV type 18 exhibits a different pattern of oncogene expression compared to other HPV types; in addition to high expression of the E7 oncogene [12], it has been suggested that HPV type 18 targets and mutates stem cell-like cells, and it has become clear that it possesses a unique mechanism in the carcinogenesis process [13].

Comprehensive analyses by the Cancer Genome Atlas (TCGA) have shown that SCC and AC differ distinctly in terms of genetic mutations, copy number abnormalities, DNA methylation, transcriptional profiles, and signaling pathway activity [14]. In SCC, mutations in genes such as CASP8, HLA class I, FBXW7, and EP300 are common, and the disease is characterized by activation of p63, the hypoxia response (HIF1A), and the MAPK signaling pathway. In contrast, AC is associated with a high frequency of mutations in ERBB2/ERBB3, KRAS, PTEN, and ARID1A, suggesting the involvement of hormone-related transcription factors (such as FOXA1/FOXA2) and estrogen signaling. Additionally, a high-methylation pattern similar to the CIMP (CpG island methylator phenotype) has been reported in AC. Campos-Parra et al. also identified 1768 differentially expressed genes between SCC and AC in the TCGA data and reported that 70 genes involved in the IL-17, JAK/STAT, and Ras signaling pathways showed differential expression [15]. It is believed that differences in molecular pathophysiology resulting from these histological types influence prognosis and response to treatment. Furthermore, in a study by Salarzaei et al., a comparison of SCC and AC identified 26 differentially expressed genes; 19 of these were overexpressed in SCC compared to AC, indicating distinct patterns of gene expression, signaling pathway activation, and immune cell infiltration between SCC and AC [16]. In AC, there are two carcinogenic pathways—HPV18-dependent and HPV18-independent (particularly GAS)—characterized by KRAS/ERBB2/ARID1A mutations and an immunosuppressive tumor microenvironment. It has been suggested that, unlike SCC, AC exhibits particularly high immune evasion capacity, and that its “immune-cold” tumor characteristics are associated with treatment resistance.

To elucidate the differences in the molecular mechanisms of carcinogenesis between SCC and AC, Kim et al. performed 10K oligonucleotide microarray analysis and signaling pathway analysis, demonstrating that SCC and AC can be clearly classified based on the expression patterns of 46 genes. They showed that immune abnormalities and PPARγ-related pathways are characteristic carcinogenic pathways in AC, whereas IGF1R-related DNA damage response pathways are characteristic in SCC [17]. Bispo et al. performed RNA sequencing analyses on HPV16-positive SCC and AC and found that the epithelial maintenance pathway, intrinsic to epithelial cells, was specifically activated in SCC, whereas the maturity-onset diabetes of the young (MODY) pathway was specifically activated in AC. These findings suggest that the carcinogenic mechanisms of the two types of cervical cancer may depend on entirely different signaling pathways [18].

### 2.2. Clinical Differences Between the “Carcinogenesis” and “Proliferation and Progression” Between SCC and AC

In AC cases, infection with HPV types 18 and 45 is common, and progression from precancerous lesions to invasive carcinoma is rapid. This rapid progression is evident from the small difference in age between the diagnosis of the precancerous lesion and that of invasive carcinoma [19]. Regarding differences in the patterns of invasion between SCC and AC, SCC is characterized by a higher rate of lymphatic invasion and regional lymph node metastasis, indicating more localized progression, whereas AC is characterized by a significantly higher rate of distant metastasis, such as metastasis to para-aortic lymph nodes, and invasion of the uterine body. Furthermore, many cases of AC present with pleural effusion or ascites, and the prognosis is considered poorer compared to SCC [20,21,22].

Santin et al. have suggested that the significantly high levels of vascular endothelial growth factor secreted by AC may induce distant metastasis [23]. Ramirez-Torres et al. reported that while SCC expresses many proteins associated with invasion and metastasis, AC expresses many proteins associated with resistance to cell death, such as RAB14 and RCN3 [24]. These reports also suggest differences in the clinical behavior of the two types.

## 3. Differences in Mechanisms of Carcinogenesis Among AC Subtypes

### 3.1. Histological Classification

The classification of adenocarcinoma has undergone significant changes following the development of the International Endocervical Adenocarcinoma Criteria and Classification (IECC), an international morphological diagnostic criteria system, in 2017 [25]. This diagnostic criteria system is a classification that categorizes AC into HPV-associated and HPV- independent types based on morphological characteristics, and it has demonstrated higher reproducibility than the conventional WHO classification. “HPV-associated adenocarcinoma” is primarily composed of usual-type adenocarcinoma and is characterized by p16 protein overexpression and HPV RNA positivity. On the other hand, “HPV- independent adenocarcinoma” includes gastric-type adenocarcinoma, clear cell carcinoma, mesonephric carcinoma, and endometrioid-like carcinoma.

This classification clarifies that “HPV- independent adenocarcinoma” is associated with a poor prognosis and plays a crucial role not only in clinical risk stratification but also as a foundation for the development of new treatments. In HPV- independent adenocarcinoma, as represented by GAS, there are many clinically advanced and recurrent cases, and these cases exhibit a poorer prognosis compared to HPV-associated adenocarcinoma. This is due to resistance to radiation and chemotherapy; however, this clinical reality underscores the need to stratify treatment selection based on histological type and etiology [26,27].

### 3.2. Differences in Mechanisms of Carcinogenesis

HPV-associated adenocarcinoma depends on E6/E7 expression resulting from persistent HPV infection. In contrast, HPV-independent adenocarcinoma exhibits carcinogenic pathways based on genetic abnormalities in genes such as TP53, KRAS, STK11, ARID1A, and PTEN; this represents a fundamental difference in mechanisms of carcinogenesis.

Usual-type adenocarcinoma, which constitutes the majority of HPV-associated adenocarcinoma, is characterized by a relatively high frequency of driver gene mutations in PIK3CA, ERBB2/ERBB3, and KRAS, against a background of genomic instability associated with the integration of HPV-DNA into the host genome [14,28,29]. Villoglandular carcinoma is also included in the group of HPV-associated adenocarcinomas; it is characterized by a high proportion of cases occurring at a younger age and cases with a favorable prognosis, and its molecular profile is similar to that of the usual type [30,31].

On the other hand, HPV-independent adenocarcinomas, as represented by GAS, exhibit characteristics that are clearly distinct from those of HPV-associated adenocarcinomas, both histopathologically and molecularly. Yi Wang et al. demonstrated through whole-exome sequencing and RNA sequencing that activation of the PI3K/AKT pathway and PIK3CA mutations are frequently observed (approximately 36%) in HPV-independent adenocarcinoma, and that this pathway plays a central role in tumor progression [32]. Furthermore, an analysis by Fernandes et al. showed that driver mutations in tumor suppressor genes such as TP53, PTEN, KRAS, ARID1A, and CTNNB1 are characteristic of HPV-independent adenocarcinoma, supporting the existence of a carcinogenic pathway independent of the HPV-dependent type [33]. Furthermore, in a large-scale genetic analysis of 68 cases of GAS, Selenica et al. revealed a high frequency of mutations in TP53, KRAS, ERBB2/3, ARID1A, and PIK3CA, and pointed out the possibility that abnormalities in cell cycle control, ERBB signaling, and ARID1A-dependent chromatin regulation may be involved in tumor formation [34].

Regarding other HPV-independent adenocarcinomas, mesonephric carcinoma exhibits KRAS (rarely NRAS) mutations in 81–100% of cases and is associated with mutations in SWI/SNF complex genes such as ARID1A, ARID1B, and SMARCA4 in approximately 60% of cases [35,36]. It is also characterized by high-frequency copy number gains in 1q (87–95%), chromosome 10 (50–79%), and chromosome 12 (approximately 74%), and exhibits a specific profile that differs molecularly and morphologically from other ACs [37]. Clear cell carcinoma is characterized by abnormalities in the Hippo pathway (WWTR1/TAZ mutations) and ARID1A and PIK3CA mutations [38,39]. Endometrioid-like carcinoma is characterized by PTEN, PIK3CA, ARID1A, and CTNNB1 mutations [40]. In serous carcinoma, TP53 mutations are the primary driver mutations, and these tumors often share characteristics similar to endometrioid carcinoma, which originates from the endometrium [41].

Figure 1 shows a schematic overview of representative molecular features distinguishing HPV-associated adenocarcinoma, HPV-independent adenocarcinoma, particularly gastric-type adenocarcinoma, and squamous cell carcinoma of the cervix. HPV-associated AC is driven by high-risk HPV infection and is characterized by recurrent alterations involving the PI3K/AKT pathway, including *PIK3CA* mutations and, in subsets of tumors, *KRAS* alterations and *PTEN* loss. In contrast, HPV-independent AC, particularly GAS, exhibits a distinct molecular landscape enriched for *TP53*, *CDKN2A*, *STK11* (LKB1), *KRAS*, and *ARID1A* alterations. ERBB2 (HER2) amplification/overexpression and CLDN18.2 expression represent potentially actionable alterations in selected GAS tumors. GAS is also characterized by aggressive biological behavior, mucinous/gastric-type differentiation, and a tendency toward an immune-cold tumor microenvironment. In SCC, high-risk HPV E6 promotes p53 degradation or suppression, whereas E7-mediated RB inactivation releases E2F and promotes cell-cycle progression. SCC additionally shows enrichment of HLA class I, *EP300*, and MAPK-related alterations and generally exhibits a more immune-infiltrated, immune-active phenotype. These subtype-specific molecular differences provide a biological rationale for histology- and biomarker-directed therapeutic strategies.

## 4. Recent Clinical Trials Expanding Treatment Options

As I have explained so far, since AC has a different biological background and treatment sensitivity compared to SCC and is associated with a poor prognosis, treatment efficacy must be reevaluated based on histological type. With the widespread adoption of molecularly targeted therapies, including immune checkpoint inhibitors, new molecularly targeted drugs are expected to improve the prognosis of AC cases; however, there are very few clinical trials specifically focused on AC, and most clinical trials include both AC and SCC.

The GOG240 trial (NCT00803062) is an international Phase III trial that evaluated the efficacy of adding the anti-VEGF antibody bevacizumab to chemotherapy (cisplatin plus paclitaxel or topotecan plus paclitaxel) for recurrent, persistent, or metastatic cervical cancer [42,43]. In this trial, the bevacizumab-combination group demonstrated a statistically significant improvement in overall survival (OS) compared to the chemotherapy-alone group in the overall population, with a median OS of 16.8 months versus 13.3 months (hazard ratio: HR 0.77, 95% CI 0.62–0.95). Approximately 30% of the enrolled patients had non-SCC, including AC and adenosquamous carcinoma (ASC); subgroup analyses indicated that the treatment effect was generally consistent across histological types (SCC vs. non-SCC). The HR for OS in AC cases alone was not explicitly stated in the paper, and the available data are limited for a statistical assessment of the treatment effect specific to AC.

KEYNOTE-A18 was an international Phase III trial that evaluated the added benefit of the anti-PD-1 antibody pembrolizumab to chemoradiotherapy (CCRT) for locally advanced cervical cancer; the primary endpoint of the trial was Event-Free Survival (EFS), and the secondary endpoint was OS. In the primary analysis, the pembrolizumab combination group showed a statistically significant improvement in EFS compared to the control group (HR 0.70, 95% CI 0.55–0.89) [44]. In the second interim analysis, OS showed a favorable trend in the combination group, with 36-month OS rates reported as 82.6% in the combination group and 74.8% in the control group (HR 0.67). Regarding histological subtype, 17% of enrolled cases were non-SCC (AC/ASC), and subgroup analysis also appears to show a trend toward better OS in the combination group (HR < 1). However, definitive hazard ratio (HR) values (EFS/OS) limited to AC alone or the non-SCC group cannot be confirmed in the publicly available data from the paper. Publication of additional analysis results limited to AC cases is desired.

The INTERLACE trial is an international, multicenter Phase III randomized controlled trial that compared a group receiving “induction chemotherapy”, consisting of carboplatin plus paclitaxel administered once weekly for 6 weeks prior to standard CCRT, with a group receiving CCRT alone in patients with locally advanced cervical cancer (FIGO 2008 IB1/IB2–IVA) [45]. In the analysis of 5-year PFS, the HR was 0.65 (95% CI 0.46–0.91), and for 5-year OS, the HR was 0.60 (95% CI 0.40–0.91); both showed a significant improvement in prognosis (IC + CCRT group: PFS 72% vs. 64%, OS 80% vs. 72%). Although AC and ASC were also included in this trial, HRs from subgroup analyses were not reported; therefore, caution is required when interpreting the treatment efficacy for non-SCC (AC/ASC) cases.

The KEYNOTE-826 trial was a Phase III randomized, double-blind study designed to evaluate whether the addition of pembrolizumab to platinum-based chemotherapy with or without the anti-VEGF antibody bevacizumab resulted in prolonged survival in patients with persistent, recurrent, or metastatic cervical cancer [46]. Approximately 20% of enrolled patients had non-SCC (AC/ASC) cases. Subgroup analysis revealed a significant improvement in prognosis in the SCC group, with an HR of 0.61 (95% CI 0.47–0.80) for OS, whereas in the non-SCC group (AC/ASC), the HR for OS was 0.76 (95% CI 0.47–1.23), indicating a trend toward improved prognosis but not reaching statistical significance [47]. These results suggest that the therapeutic effect may be reduced in AC cases compared to SCC cases, and they raise important considerations for the design of future clinical trials.

EMPOWER-Cervical 1 (GOG-3016/ENGOT-cx9; NCT03257267) was an international Phase III randomized trial enrolling patients with recurrent or metastatic cervical cancer following platinum-based chemotherapy. The trial compared the PD-1 inhibitor cemiplimab monotherapy with chemotherapy (including pemetrexed, gemcitabine, topotecan, irinotecan, and vinorelbine), with OS as the primary endpoint [48]. Among the enrolled patients, SCC cases accounted for approximately 77.8%, while non-SCC (AC/ASC) cases accounted for approximately 22.2%. The median OS was 11.7 months in the cemiplimab group and 8.5 months in the chemotherapy group, with an HR of 0.67 (95% CI 0.56–0.80, *p* < 0.00001). A trend toward survival prolongation was also reported in the non-SCC group, with published results showing a median OS of 13.3 months vs. 7.0 months (HR 0.56; 95% CI 0.36–0.85; *p* < 0.005). Cemiplimab demonstrated a significant improvement in survival even in non-SCC (AC/ASC) cases; however, detailed results specific to AC cases, excluding ASC, have not been published.

The innovaTV 301 trial (ENGOT-cx12/GOG-3057; NCT04697628) is an international Phase III trial comparing the antibody-drug conjugate (ADC) tisotumab vedotin (TV) with single-agent chemotherapy (topotecan, vinorelbine, gemcitabine, irinotecan, or paclitaxel) in patients with recurrent or metastatic cervical cancer following platinum-based therapy [49]. The primary endpoint was OS, and the secondary endpoints were PFS and objective response rate (ORR). In terms of histology, approximately 60% of enrollees had SCC, and approximately 40% had non-SCC (AC/ASC). In the final results, tisotumab vedotin significantly prolonged OS compared to chemotherapy (median 11.5 vs. 9.5 months, HR 0.70) and demonstrated superiority in PFS (4.2 vs. 3.5 months) and ORR (17.8% vs. 5.2%); however, the HR for the non-SCC (AC/ASC) was not reported.

As noted above, there are very few studies that have analyzed the therapeutic efficacy of new treatments specifically in AC cases, and the evidence is clearly insufficient.

## 5. Ongoing Clinical Trials of Novel Therapies for Cervical Cancer Including AC

Ongoing clinical trials of new treatments for cervical cancer including AC are shown in Table 1. Among the clinical trials listed in Table 1, the following treatments are considered highly likely to become standard of care within the next few years.

Sacituzumab tirumotecan is an ADC targeting TROP2. The TroFuse-020 trial is investigating whether the ADC therapy can improve survival compared with standard second-line chemotherapy in recurrent cervical cancer [50]. The TroFuse-036 trial aims to establish whether maintenance ADC therapy can prolong disease control after induction chemo-immunotherapy, addressing the high rate of progression within 1 year after KEYNOTE-826–based treatment [51].

In combination therapy with tisotumab vedotin and other molecularly targeted agents, the goal of NCT03786081 (innovaTV205/ENGOT-cx8/GOG-3024) is to evaluate whether combining tisotumab vedotin with immune checkpoint inhibitors or anti-VEGF therapy can improve response rates beyond those seen with ADC monotherapy, and an ORR of 35–55% has been achieved [52]. In addition, a clinical trial is underway evaluating the combination of CRB-701 (SYS6002), an ADC targeting Nectin-4, and pembrolizumab [53].

NCT04405349 is a Phase II trial investigating the efficacy of combination therapy with VB10.16, an HPV therapeutic vaccine, and atezolizumab in patients with HPV16-positive recurrent or metastatic cervical cancer [54]. The trial aims to enhance HPV-specific T-cell immunity and verify synergistic effects with PD-L1 inhibitors.

**Table 1:** Ongoing clinical trials of novel therapies for cervical cancer including AC.

Clinical Trial (NCT)	Phase	Patient Population	Investigational Treatment	Target/Mechanism	Trial Concept/Objective	Primary Endpoint	Key Publications
NCT06459180 (TroFuse-020/GOG-3101/ENGOT-cx20)	III	Patients with recurrent or metastatic cervical cancer who have progressed after platinum-based chemotherapy (including squamous cell carcinoma, adenocarcinoma, and adenosquamous carcinoma)	Sacituzumab tirumotecan	TROP2-targeted ADC	Compare TROP2-targeted ADC with physician’s-choice chemotherapy in second-line treatment.	OS, PFS	Salani R et al., *J Gynecol Cancer*, 2025. [50]
NCT07216703 TroFuse-036 (GOG-3123/ENGOT-cx22)	III	Patients with PD-L1-positive persistent, recurrent, or stage IVB cervical cancer who have not progressed after first-line pembrolizumab plus platinum-based chemotherapy ± bevacizumab	Sacituzumab tirumotecan + pembrolizumab ± bevacizumab	TROP2 ADC + PD-1 blockade	Evaluate ADC-based maintenance therapy following first-line chemo-immunotherapy.	PFS, OS	Slomovitz BM et al., *J Clin Oncol*, 2026. [51]
NCT03786081 (innovaTV205/ENGOT-cx8/GOG-3024)	Ib/II	Patients with recurrent or metastatic cervical cancer after one or more prior systemic therapies	Tisotumab vedotin + pembrolizumab/carboplatin/bevacizumab	Tissue Factor-targeted ADC	Evaluate combination strategies to improve efficacy beyond ADC monotherapy.	ORR, Safety	Vergote et al., *J Clin Oncol.* 2023. [52]
NCT04246489	II	Patients with persistent, recurrent, or metastatic cervical cancer previously treated with platinum-based chemotherapy	Bintrafusp alfa (M7824)	Bifunctional TGF-β trap + PD-L1 inhibitor	Simultaneously inhibit TGF-β-mediated immune suppression and PD-L1 signaling to enhance antitumor immunity.	ORR	Birrer et al., *JAMA Oncology*, 2024. [55]
NCT06265727/CRB-701 (SYS6002)	I/II	Patients with advanced solid tumors, including cervical cancer, refractory to standard treatment	CRB-701	Nectin-4-targeted ADC	Evaluate Nectin-4 as a novel therapeutic target.	ORR, Safety	Ciuleanu T-E et al., *J Clin Oncol*, 2026. [53]
NCT04405349 VB10.16 + Atezolizumab	IIa	Patients with HPV16-positive recurrent or metastatic cervical cancer	Therapeutic HPV DNA vaccine + atezolizumab	HPV16 E7 vaccine + PD-L1 blockade	Enhance HPV-specific immune responses in recurrent disease.	ORR, Safety	Hillemanns et al., *J Immunother Cancer*. 2025. [54]
NCT03108495 LN-145 (Lifileucel)	II	Patients with recurrent cervical cancer progressing after immune checkpoint inhibitor therapy	Tumor-infiltrating lymphocyte (TIL) therapy	Adoptive cell therapy	Evaluate personalized TIL therapy in immunotherapy-resistant disease.	ORR, Duration of Response	Stevanović et al., *Clin Cancer Res*, 2019. [56]
NCT05686226 HPV-specific TCR-T Cell Therapy	II	Patients with HPV16-positive recurrent or metastatic cervical cancer	Engineered TCR-T cells	HPV16 E6/E7-specific T-cell therapy	Develop antigen-specific cellular immunotherapy for HPV-driven tumors.	Safety, ORR	Nagarsheth NB et al., *Nat Med*, 2021. [57]

## 6. New Ongoing Clinical Trials Targeting Only AC

Ongoing clinical trials focused on AC are shown in Table 2. Although the number is very small, several treatments based on the molecular pathophysiology specific to AC are currently in development. In particular, for HPV-independent GAS, the NCT06654011 trial is underway, evaluating a combination therapy of IN10018 and nab-paclitaxel [58]. The first dedicated molecular-targeted trial exclusively for GAS. Based on evidence that GAS frequently exhibits FAK pathway activation, immune resistance, and poor responsiveness to conventional therapies.

Clinical trials or HER2-targeted ADCs include the HER2-directed Basket Trials (Trastuzumab deruxtecan) and the Disitamab Vedotin Basket Studies (RC48) [59,60]. AC cases, especially GAS, show substantially higher HER2 amplification rates than SCC, making HER2 a rational therapeutic target.

Figure 2 represents a schematic representation of major oncogenic signaling pathways and potentially actionable molecular targets relevant to cervical adenocarcinoma and squamous cell carcinoma. Receptor tyrosine kinases, including HER2 (ERBB2), ERBB3, EGFR, and ROS1, converge on downstream PI3K–AKT–mTOR and RAS–RAF–MEK–ERK/MAPK signaling pathways. Loss of negative regulators, including *PTEN* and *STK11* (LKB1), may further promote pathway activation, proliferation, therapeutic resistance, and an immune-cold phenotype. *KRAS* alterations activate MAPK signaling, whereas *ARID1A* loss results in chromatin-remodeling dysfunction and may create genomic or transcriptional vulnerabilities. Potential therapeutic approaches include HER2-directed antibody–drug conjugates, PI3K/AKT/mTOR pathway inhibitors, and FAK inhibitors. Actionable cell-surface antigens include CLDN18.2, TROP2, tissue factor, and Nectin-4, providing potential targets for antibody- or ADC-based therapies. CLDN18.2-directed approaches may be particularly relevant to gastric-type adenocarcinoma. VEGF-mediated angiogenesis remains an established therapeutic target, while immune checkpoint inhibition is clinically relevant across cervical cancer subtypes, although its biological rationale may be particularly strong in immune-infiltrated SCC. Solid arrows indicate relatively established biological associations, dashed arrows indicate emerging, less-established, or context-dependent associations, and T-bars indicate inhibitory interactions.

## 7. Future Prospects for New Treatment Strategies for AC

Jin Li et al. analyzed somatic mutations and biomarkers associated with the immune response using tumor tissue and liquid biopsy samples to identify gene mutations specific to SCC and AC. While SCC was associated with a high tumor mutational burden (TMB), AC was characterized by a high prevalence of STK11 mutations; furthermore, genetic alterations specific to AC were significantly associated with reduced infiltration of B cells, T cells, and dendritic cells. These findings suggest that molecularly targeted therapies may be more effective in AC patients than in SCC patients [62].

Li et al. used single-cell RNA sequencing to elucidate the tumor heterogeneity and differences in the tumor microenvironment between SCC and AC [63]. In SCC, epithelial–mesenchymal transition (EMT), the hypoxic response, and inflammatory pathways were activated; cytotoxic CD8 T cells, activated NK cells, and MHC-II-high-expressing macrophages were abundant in the stroma, indicating characteristics consistent with a stronger immune response. In contrast, in AC, cell cycle pathways were predominant, with a high abundance of naive T cells and Tregs, indicating an immunosuppressive environment. These findings suggest that immunotherapy is likely to be effective in SCC patients, whereas treatments targeting metabolic and cell cycle pathways or fibroblasts hold promise for AC patients.

Furthermore, Machida et al. reported that mutations in ROS1, EGFR, PIK3CA, and other genes are frequently observed in AC patients. In particular, cases with a high number of ROS1 mutations showed increased recurrence rates and reduced survival rates. This suggests that ROS1 mutations may serve as a marker of poor prognosis in AC and points to the potential of tyrosine kinase inhibitors for treating ROS1-mutated AC patients [64].

Figure 3 represents therapeutic approaches categorized as established, emerging, or investigational according to their current clinical applicability in cervical cancer, with particular consideration of cervical adenocarcinoma. Established therapies include the tissue factor–directed ADC tisotumab vedotin, the anti-VEGF antibody bevacizumab, and immune checkpoint inhibitors such as pembrolizumab, cemiplimab, and nivolumab. Emerging biomarker-directed strategies include HER2-targeted ADCs, represented by trastuzumab deruxtecan (T-DXd) and disitamab vedotin (RC48), which may be particularly relevant to HER2-expressing or HER2-amplified tumors. Investigational approaches include CLDN18.2-targeted therapies such as zolbetuximab and CLDN18.2-directed ADCs; TROP2-directed ADCs, including sacituzumab govitecan; Nectin-4-targeted ADC strategies; FAK inhibitors such as defactinib and IN10018; inhibitors of the PI3K/AKT/mTOR pathway, including alpelisib, inavolisib, and everolimus; and ROS1 tyrosine kinase inhibitors such as crizotinib and entrectinib in selected rare molecularly defined cases. The classification reflects the current level of clinical applicability in cervical cancer and should not be interpreted as indicating equivalent levels of regulatory approval or clinical evidence across individual agents, histologic subtypes, or geographic regions.

## 8. Conclusions

Cervical adenocarcinoma is a distinct entity that differs markedly from SCC in terms of epidemiology, pathogenesis, molecular profile, tumor microenvironment, and clinical behavior. While AC is strongly associated with HPV type 18, the existence of HPV-independent adenocarcinomas, such as GAS and mesonephric-type carcinoma, has become evident, and these exhibit distinct molecular characteristics even within the histological subclassification of AC. Multidimensional omics analyses, including those from the TCGA, have shown that while CASP8 mutations and HIF1A/MAPK activation are predominant in SCC, AC is characterized by ERBB2/ERBB3, KRAS, ARID1A, and PTEN mutations, as well as a CIMP-like hypermethylation phenotype. In the immune microenvironment, AC also exhibits immunosuppressive and “immune-cold” characteristics, which are thought to underlie the limited efficacy of immunotherapy alone. Cervical adenocarcinoma is not merely a “non-squamous cell carcinoma” but a group of tumors that should be understood as a distinct disease entity.

In the future, further improvements in treatment outcomes are expected through stratification based on HPV association and molecular subtypes, as well as the development of personalized therapies targeting the immune microenvironment and metabolic pathways.

## Figures and Tables

**Figure 1:**
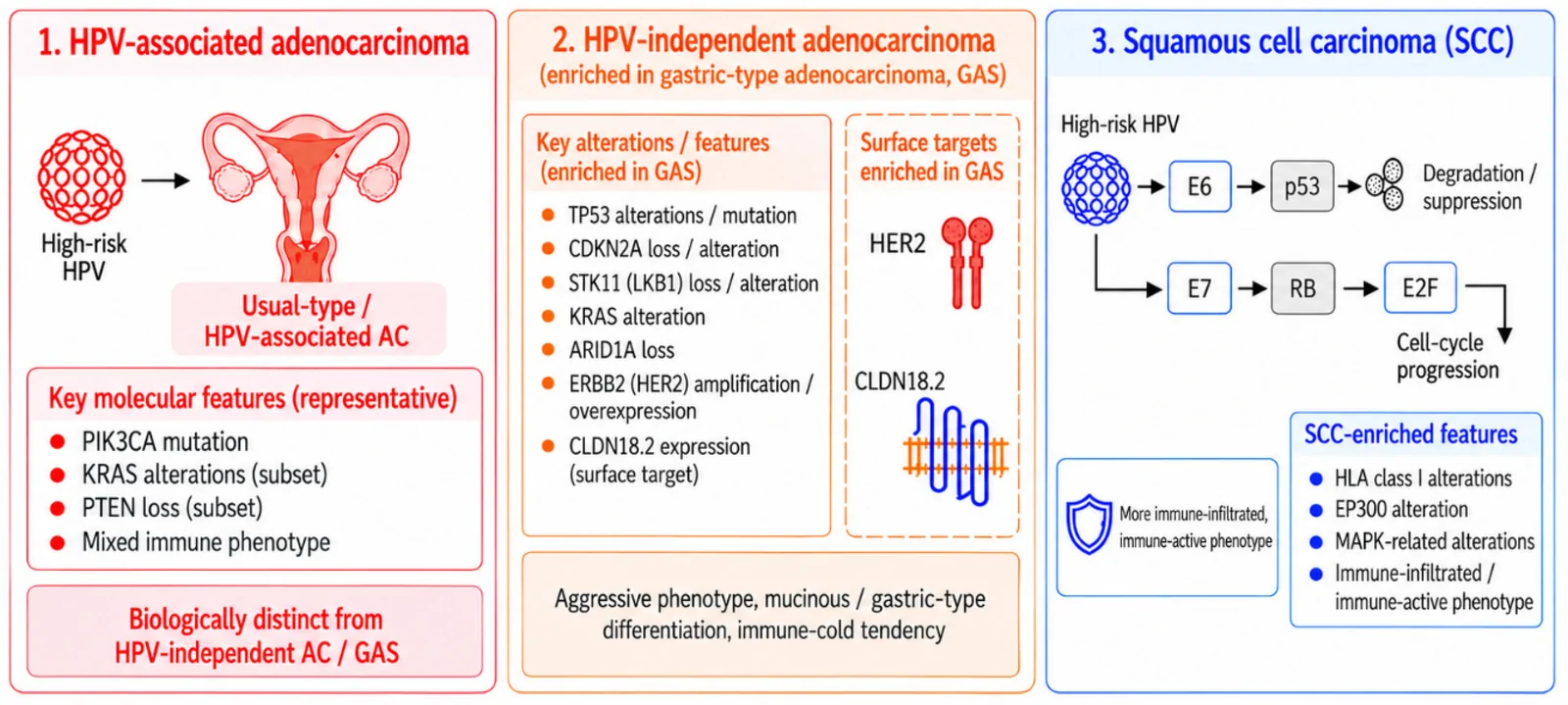
Distinct molecular landscapes and therapeutic vulnerabilities of cervical adenocarcinoma and squamous cell carcinoma. Schematic overview of representative molecular features of HPV-associated adenocarcinoma, HPV-independent adenocarcinoma, particularly gastric-type adenocarcinoma (GAS), and squamous cell carcinoma (SCC). The figure highlights subtype-specific genomic alterations, immune phenotypes, and potentially actionable targets, including HER2 and CLDN18.2. Abbreviations: AC, adenocarcinoma; GAS, gastric-type adenocarcinoma; SCC, squamous cell carcinoma; HPV, human papillomavirus; HLA, human leukocyte antigen; MAPK, mitogen-activated protein kinase. This figure was created by modifying an original draft—newly produced based on the authors’ scientific insights—using OpenAI. The authors verified the scientific content included in the figure by cross-referencing relevant literature.

**Figure 2:**
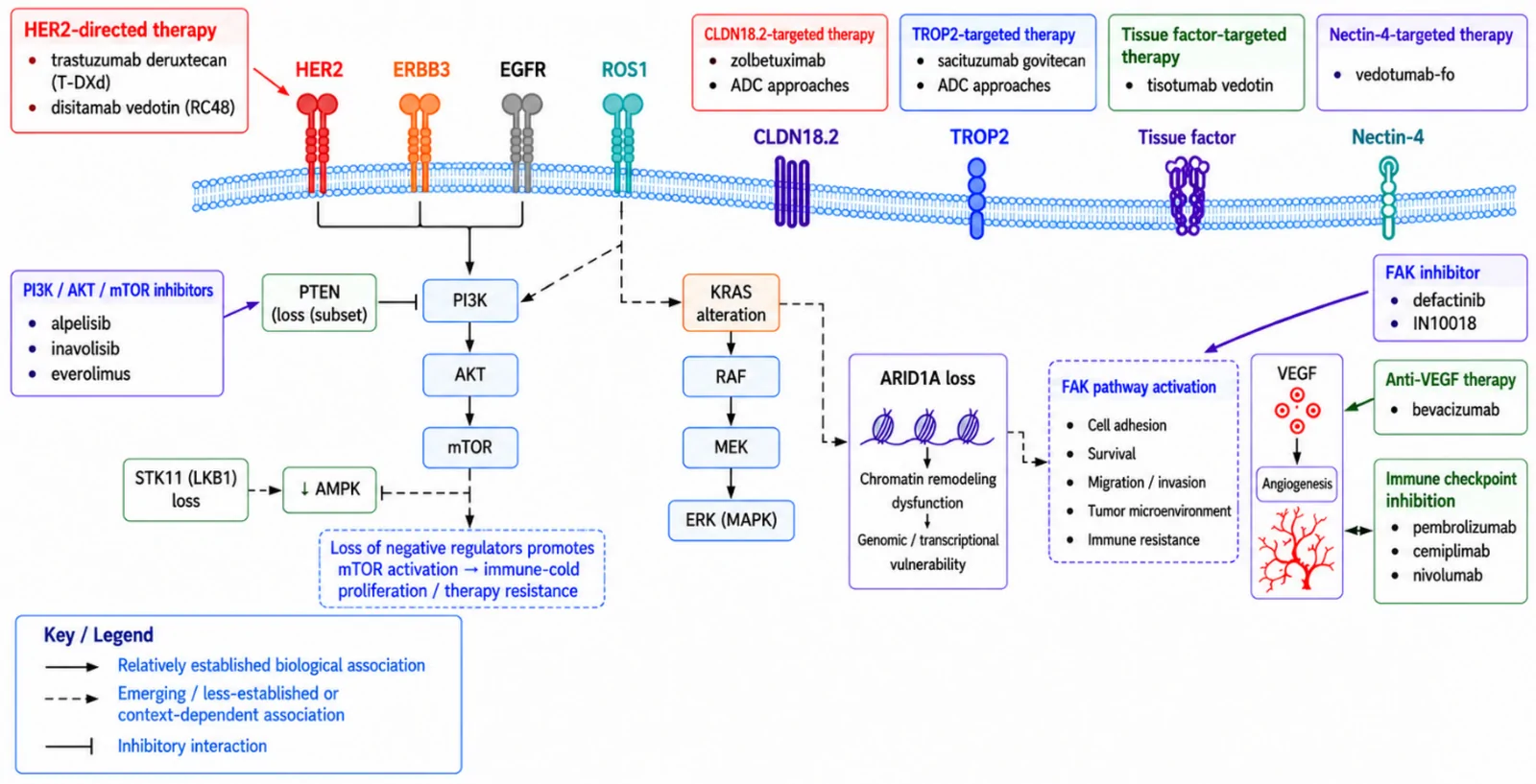
Molecular pathways and therapeutic targets in cervical cancer. Overview of major oncogenic pathways and potentially actionable targets in cervical cancer. Key alterations involving HER2, PI3K–AKT–mTOR, RAS–MAPK, ARID1A, and FAK pathways are shown together with cell-surface targets, angiogenesis, and immune checkpoint pathways. Solid and dashed arrows indicate relatively established and emerging/context-dependent associations, respectively. Abbreviations: AC, adenocarcinoma; GAS, gastric-type adenocarcinoma; SCC, squamous cell carcinoma; ADC, antibody–drug conjugate; HER2, human epidermal growth factor receptor 2; EGFR, epidermal growth factor receptor; PI3K, phosphatidylinositol 3-kinase; AKT, protein kinase B; mTOR, mechanistic target of rapamycin; MAPK, mitogen-activated protein kinase; FAK, focal adhesion kinase; VEGF, vascular endothelial growth factor; TKI, tyrosine kinase inhibitor. This figure was created by modifying an original draft—newly produced based on the authors’ scientific insights—using OpenAI. The authors verified the scientific content included in the figure by cross-referencing relevant literature.

**Figure 3:**
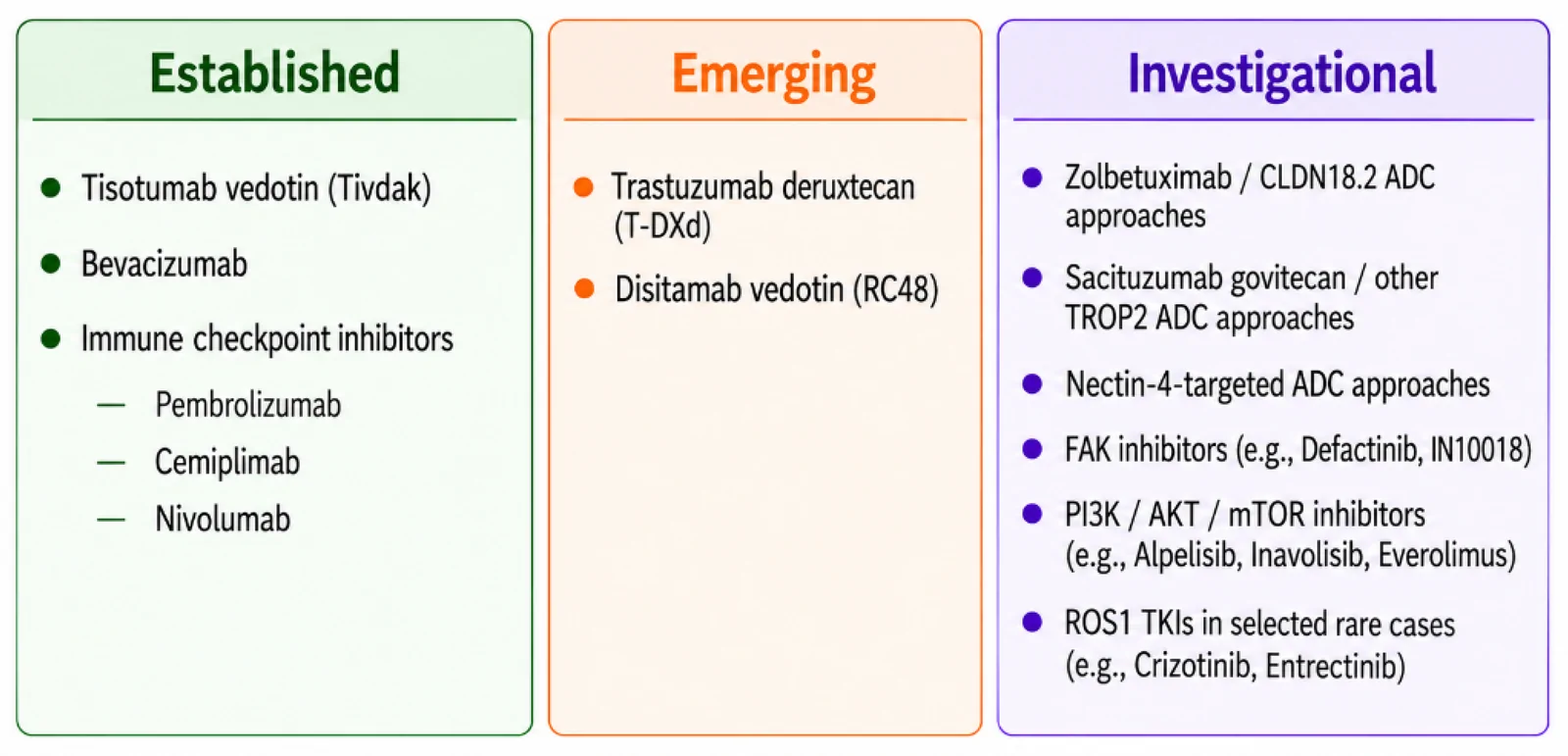
Drug classification by clinical applicability in cervical cancer. Therapeutic approaches are categorized as established, emerging, or investigational according to their current clinical applicability in cervical cancer. The figure summarizes approved systemic therapies and emerging biomarker-directed strategies, including ADCs, pathway-targeted agents, and molecularly selected therapies. The classification reflects the current level of clinical applicability in cervical cancer and should not be interpreted as indicating equivalent levels of regulatory approval or clinical evidence across individual agents, histologic subtypes, or geographic regions. Abbreviations: ADC, antibody–drug conjugate; CLDN18.2, claudin 18.2; FAK, focal adhesion kinase; PI3K, phosphatidylinositol 3-kinase; AKT, protein kinase B; mTOR, mechanistic target of rapamycin; ROS1, ROS proto-oncogene 1 receptor tyrosine kinase; TKI, tyrosine kinase inhibitor; T-DXd, trastuzumab deruxtecan.

**Table 2:** New ongoing clinical trials targeting only AC.

Clinical Trial	Phase	Patient Population	Investigational Treatment	Target/Mechanism	Trial Concept/Objective	Primary Endpoint	Key Publications
NCT06654011 (GIFT Study)	II	Patients with metastatic, recurrent, or persistent gastric-type endocervical adenocarcinoma (HPV-independent subtype) after at least one prior systemic chemotherapy regimen	IN10018 + nab-paclitaxel + cadonilimab	FAK inhibitor + PD-1/CTLA-4 bispecific antibody	First prospective trial dedicated exclusively to gastric-type endocervical adenocarcinoma (GEA). Based on translational evidence that stromal FAK activation contributes to immune evasion and resistance to chemotherapy in GEA.	ORR	Zhao Y et al., *J Clin Oncol*, 2026. [58]
NCT04482309 HER2-directed Basket Trials (DESTINY-PanTumor02, institutional HER2 studies)	II	Patients with HER2-positive cervical adenocarcinoma (included within pan-tumor cohorts)	Trastuzumab deruxtecan (T-DXd)	HER2-targeted antibody-drug conjugate	Evaluate HER2-targeted therapy in HER2-overexpressing rare cancers, including cervical adenocarcinoma. Cervical adenocarcinoma—particularly gastric-type tumors—has a substantially higher HER2 amplification rate than squamous carcinoma.	ORR	Meric-Bernstam F et al., *J Clin Oncol*, 2024. [59]
NCT04965519 Disitamab Vedotin (RC48) Basket Studies	II	Patients with HER2-expressing cervical adenocarcinoma	Disitamab vedotin (RC48)	HER2-targeted antibody-drug conjugate	Explore HER2-directed ADC therapy in rare HER2-positive tumors, including cervical adenocarcinoma.	ORR	Yuan G et al., *J Clin Oncol*, 2024. [60]
NCT04683939/BNT141-01 Claudin18.2 Exploratory Programs	Early phase	Selected patients with gastric-type cervical adenocarcinoma expressing Claudin18.2	Experimental anti-CLDN18.2 therapies	Claudin18.2	Based on molecular similarity between gastric-type cervical adenocarcinoma and gastrointestinal adenocarcinoma. Currently limited to exploratory or basket studies.	Safety, ORR	N Yasutake et al., *Histopathology*, 2025. [61]

## Data Availability

No new data were created or analyzed in this study. Data sharing is not applicable to this article.
